# Targeting the Notch signaling pathway: molecular mechanisms and therapeutic strategies for cardiac repair after myocardial infarction

**DOI:** 10.3389/fcvm.2025.1669362

**Published:** 2025-11-21

**Authors:** ZhiCheng Zhang, FengQun Xie, Jie Cheng, Xinyue Liu, Xinran Wang, Jun Long, QiMao Feng, Dongping Yuan

**Affiliations:** 1 The People’s Hospital of Yuhuan, Yuhuan, China; 2 Shanghai Municipal Hospital of Traditional Chinese Medicine, Shanghai University of Traditional Chinese Medicine, Shanghai, China; 3 General Practice Department, Shanghai Tongji Hospital, Shanghai, China; 4 School of Pharmacy, Nanjing University of Chinese Medicine, Nanjing, China

**Keywords:** Notch signaling pathway, myocardial infarction, cardiac repair, cardiomyocyte regeneration, angiogenesis

## Abstract

Myocardial infarction (MI) is a leading cause of death globally and is characterized by extensive cardiomyocyte death, fibrosis, and diminished cardiac function, leading to heart failure. The limited regenerative capacity and excessive fibrosis of the heart highlight the need for effective therapeutic strategies. The Notch signaling pathway, known for its role in cell fate determination and tissue repair, is transiently activated after MI in cardiomyocytes, endothelial cells, and smooth muscle cells. This activation modulates cardiac repair by reducing oxidative stress and apoptosis, regulating inflammation, promoting angiogenesis, and inhibiting fibrosis. Recent research has focused on targeting the Notch pathway to increase myocardial regeneration and angiogenesis via the use of gene therapy, small-molecule regulators, and cell-based therapies. For example, delivering Notch ligands through hydrogels has yielded promising results in preclinical studies, enhancing cardiac function and promoting angiogenesis. This review examines the molecular mechanisms by which Notch signaling influences cardiac repair post-MI. We also discuss its specific roles in cardiomyocyte regeneration, fibrosis inhibition, and angiogenesis enhancement. Additionally, this study evaluated the therapeutic potential of Notch pathway modulation, addressing clinical translation challenges, safety concerns, and the importance of personalized treatment strategies. Future research directions include leveraging gene editing and nanotechnology-based drug delivery to improve the efficacy and safety of Notch-targeted therapies for cardiovascular diseases.

## 1 Introduction

**Figure 1:**
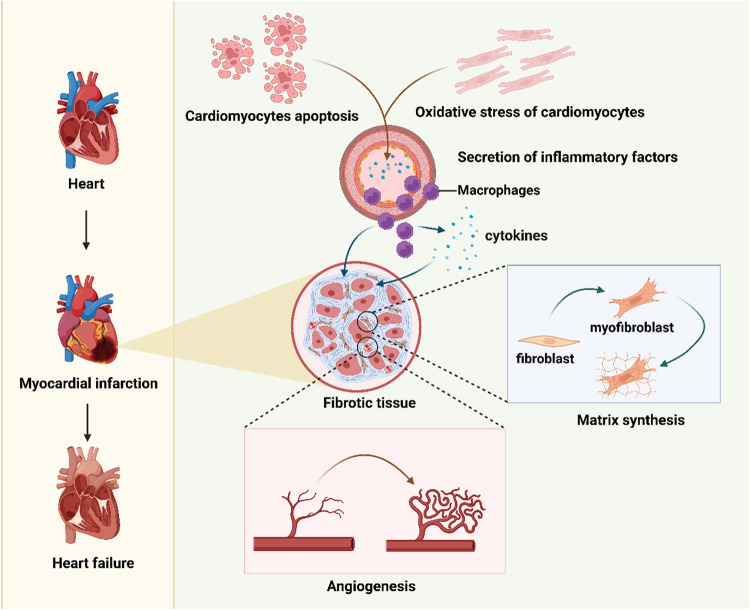
An overview of the mechanisms of cardiac pathological changes following myocardial infarction. Figure created in BioRender.com.

Myocardial infarction (MI) is a leading cause of mortality and disability worldwide (1, 2). Globally, approximately 17.9 million people die from cardiovascular diseases each year, among them, 85% are associated with MI and stroke (3). The primary pathological mechanisms of MI include extensive cardiomyocyte death (4), fibrosis triggered by ischemic injury (5–8), and the subsequent progressive loss of cardiac function, which may eventually lead to heart failure (HF) (9, 10) (Figure 1). Although the heart has a certain capacity for self-repair after injury, the regenerative potential of adult cardiomyocytes is extremely limited (11, 12). The repair process is often accompanied by excessive fibrosis. This fibrosis further impedes the restoration of cardiac function.

After a myocardial infarction (MI), post-MI cardiac remodeling describes a complex series of molecular, cellular, and interstitial changes that modify the heart's size, shape, structure, and function (13). Initially, this process acts as an adaptive response to the initial injury and the resulting hemodynamic load, with the aim of preserving cardiac output—but in most cases, it turns out to be maladaptive, leading to progressive ventricular dilation, structural distortion, and systolic dysfunction. As a key factor shaping post-MI prognosis, it ultimately contributes to HF and malignant arrhythmias. Pathologically, this remodeling process unfolds in three distinct phases: the inflammatory, proliferative, and maturation phases (13, 14). During the inflammatory phase, a controlled inflammatory response is critical for clearing damaged tissue; yet excessive or prolonged inflammation will worsen the injury and promote adverse remodeling, and the myocardium also becomes extremely vulnerable at this stage, carrying a high risk of cardiac rupture. The proliferative phase focuses on forming a mechanically stable and compliant scar, but dysregulated repair disrupts this goal—causing an imbalance in collagen types, disorganized fiber alignment, and the development of a stiff, non-compliant scar. What's more, fibrosis extends to the border and remote myocardial zones (referred to as reactive fibrosis), which impairs ventricular compliance and electrical conduction, laying the foundation for subsequent occurrence of HF and arrhythmias. The maturation phase, meanwhile, holds the key to long-term patient outcomes: maladaptive remodeling in this phase means continued progressive dilation and declining cardiac function, eventually leading to clinical HF, whereas adaptive remodeling involves the stabilization or even reversal of ventricular size and improved cardiac function.

As a highly conserved intercellular communication mechanism, the Notch signaling pathway plays crucial roles in embryonic development, cell fate determination, and tissue repair (15). In the heart, Notch1 is expressed in cardiomyocytes, endothelial cells, and smooth muscle cells (16, 17). Studies have shown that following MI, the Notch1 signaling pathway is transiently activated, regulating cardiac repair and regeneration through multiple mechanisms. These mechanisms include the suppression of oxidative stress, a reduction in apoptosis, the modulation of inflammatory responses, the promotion of angiogenesis, and the inhibition of fibrosis (18–23).

In recent years, therapeutic strategies targeting the Notch signaling pathway have attracted increasing research interest. These strategies aim to activate or inhibit the Notch pathway through gene therapy, small molecule regulators, and cell-based therapies to increase myocardial regeneration and angiogenesis (24). For example, intramyocardial injection of hydrogels containing Notch ligands has been shown to significantly improve cardiac function and promote angiogenesis, demonstrating the potential of targeting the Notch pathway in MI treatment (3).

In summary, the Notch signaling pathway plays a crucial regulatory role in cardiac repair following MI, suggesting new therapeutic avenues for related cardiovascular diseases. This review delves into the molecular mechanisms by which Notch signaling modulates post-MI cardiac repair, analyzes its specific roles in cardiomyocyte regeneration, the inhibition of fibrosis, the promotion of angiogenesis, and the therapeutic potential of targeting the Notch pathway in myocardial repair.

## 2 Pathological processes and repair mechanisms after MI

### 2.1 Cardiomyocyte apoptosis and necrosis

Extensive cardiomyocyte death is a critical pathological process following MI (25). Cell death can occur via programmed apoptosis or necrosis, which is often accompanied by an inflammatory response. Necrosis can either be nonprogrammed or occur through regulated processes such as necroptosis (26).

Apoptosis is regulated by several molecular pathways, with the mitochondrial and death receptor pathways being the most prominent. In the mitochondrial pathway, intracellular stress (e.g., oxidative stress or DNA damage) alters the permeability of the mitochondrial outer membrane by regulating Bcl-2 family proteins, leading to the release of cytochrome c into the cytosol. This release subsequently activates caspase-9, which in turn activates caspase-3, ultimately inducing apoptosis (27).

Moreover, oxidative stress exacerbates cellular damage by generating reactive oxygen species (ROS). ROS can directly attack membrane lipids and mitochondrial DNA, further promoting mitochondrial dysfunction and accelerating apoptosis (28).

On the other hand, necrosis is a nonprogrammed form of cell death typically characterized by cell membrane rupture and the release of intracellular contents, triggering a robust inflammatory response. During MI, oxygen deprivation leads to ATP depletion, impairing the ability of the cell to maintain ionic gradients and equilibrium, ultimately causing cell membrane disruption. Necrotic cells release endogenous danger-associated molecular patterns such as heat shock proteins, ATP, and high-mobility group box 1, which bind to Toll-like receptors to activate the innate immune system and trigger an acute inflammatory response (29).

### 2.2 Cardiac fibrosis and scar formation

Fibrosis is a central process in MI repair. Following the clearance of necrotic cells, the heart forms scar tissue to replace lost cardiomyocytes. However, the cellular and molecular mechanisms involved in this process often have long-term effects on cardiac function (30).

During post-myocardial infarction (post-MI) fibrosis, the pathological process involves multiple molecules and signaling pathways, with transforming growth factor-beta (TGF-β) serving as a primary driver while other mediators also play critical regulatory roles. In response to damage signals, fibroblasts are activated and recruited to the injured area, and TGF-β is one of the key initiating signals in this process (31, 32). Notably, cardiac fibroblast-specific interleukin-11 (IL-11) signaling has emerged as a critical and more direct fibrotic mediator than TGF-β in some contexts: IL-11 drives ERK-dependent autocrine signaling in cardiac fibroblasts, which further induces myofibroblast transformation and extracellular matrix (ECM) production (33).

Beyond IL-11, pro-inflammatory cytokines also contribute to fibrotic progression: interleukin-1β (IL-1β) and tumor necrosis factor-alpha (TNF-α) promote fibroblast proliferation and enhance the expression of matrix metalloproteinases (MMPs), thereby regulating ECM turnover and amplifying pro-fibrotic signaling (34). Additionally, monocyte chemoattractant protein-1 (MCP-1/CCL2) participates in fibrosis by recruiting monocytes/macrophages to the injury site; these recruited cells then secrete pro-fibrotic factors such as TGF-β, forming a positive feedback loop to perpetuate the fibrotic response (35).

Apart from cytokines, classic signaling pathways also play pivotal roles in post-MI fibrosis. The canonical Wnt/β-catenin pathway exerts a crucial regulatory effect through β-catenin stabilization and nuclear translocation: once translocated into the nucleus, β-catenin interacts with T cell factors to modulate the expression of pro-fibrotic genes, thereby promoting fibrotic progression (36). Meanwhile, all members of the platelet-derived growth factor (PDGF) family (including PDGF-AA, -BB, -AB, -CC, and -DD) contribute to fibroblast activation by binding to PDGFR-α and PDGFR-β receptors; this binding activates downstream signaling cascades such as JAK/STATs, PI3K/AKT, and RAS/MAPK pathways, further amplifying the fibrotic response. Importantly, these pathways do not act independently but exhibit complex cross-pathway integration: for instance, PDGF-D can form positive feedback loops with TGF-β, which significantly enhances fibrotic responses in both infarcted and non-infarcted myocardial regions (37, 38).

At the site of post-MI injury, activated fibroblasts further differentiate into myofibroblasts—cells with a high capacity to synthesize and secrete ECM components, particularly type I and type III collagen (39). Studies have confirmed that myofibroblasts proliferate rapidly in the infarcted region: during the early phase of post-MI repair, their density reaches up to 3.5 times that of normal heart tissue, significantly contributing to ECM accumulation (40).

Mechanistically, upon binding to its specific receptor, TGF-β activates the downstream SMAD signaling pathway, which directly promotes collagen synthesis and ECM deposition (41, 42). Concurrently, TGF-β inhibits the activity of MMPs, a family of enzymes responsible for ECM degradation; this dual effect (enhancing synthesis while suppressing degradation) further accelerates ECM accumulation (43). However, excessive fibrosis disrupts normal cardiac structure and function: it leads to increased cardiac stiffness and impairs both contractile and diastolic capacity, which can ultimately progress to HF (44).

Preclinical studies have successfully reduced fibrosis in the infarcted area and significantly improved cardiac function by inhibiting the TGF-β signaling pathway through gene-editing techniques (26). These findings offer new insights into future molecular targeted therapies aimed at regulating fibrosis.

### 2.3 Inflammatory response and cardiac remodeling

The inflammatory response following MI is essential for the repair of damaged tissues (45). Its primary function is to remove necrotic cardiomyocytes and tissue debris, thereby creating a favorable environment for cardiac regeneration and repair (46). However, excessive or prolonged inflammation can lead to pathological cardiac remodeling and exacerbate cardiac dysfunction (47).

Studies using mouse models have shown that neutrophils rapidly accumulate in the infarcted region, peaking approximately 3 h post-MI, where they begin clearing necrotic tissue (48). Neutrophils are the first immune cells recruited to the injured area within 30 min to a few hours post-MI. They exert their effects by releasing large quantities of ROS and proteolytic enzymes, such as MMPs, to degrade necrotic tissue, paving the way for subsequent macrophage infiltration.

After the clearance phase by neutrophils, which typically lasts for 2–3 days, macrophages become the dominant cell type in the injured area and differentiate into two distinct subtypes with opposing functions: proinflammatory M1 macrophages and anti-inflammatory M2 macrophages (49–51). M1 macrophages release proinflammatory cytokines, such as TNF-α and interleukin-6, to amplify the inflammatory response and ensure thorough clearance of debris in the injured area (47). In contrast, M2 macrophages become predominant during the later stages of inflammation, where they secrete anti-inflammatory cytokines, such as interleukin-10, to promote tissue repair and suppress inflammation (27).

The inflammatory response is further regulated by chemokines and their receptors. For example, the CCR2/CCL2 axis plays a critical role in macrophage recruitment, whereas the CX3CR1/CX3CL1 pathway is pivotal for macrophage polarization toward an anti-inflammatory phenotype (29). The precise regulation of these signaling pathways determines the intensity and duration of the inflammatory response. However, if the inflammatory response is not effectively resolved, the heart undergoes pathological remodeling. At this stage, the ventricular wall begins to thin, the heart chamber gradually dilates, and both the geometry and function of the heart undergo significant alterations.

Talman and Ruskoaho reported that chronic inflammation following MI accelerates myocardial fibrosis, leading to a substantial increase in left ventricular end-diastolic volume (LVEDV). Uncontrolled inflammatory responses can result in an increase in the LVEDV from 80 mL to approximately 120–130 mL, representing a 40%–60% increase, which significantly impacts the pumping efficiency of the heart and may ultimately lead to HF (30).

One of the main characteristics of this pathological remodeling is increased fibrosis and stiffness of the ventricular wall accompanied by left ventricular dilation. This geometric alteration limits the ability of the heart to contract and relax, resulting in a decline in the ejection fraction and a reduced capacity to effectively pump blood. Therefore, the duration and intensity of the inflammatory response are critical for the outcome of cardiac repair, and early intervention to modulate excessive inflammation is considered key for minimizing long-term cardiac dysfunction.

### 2.4 Angiogenesis and repair mechanisms

#### 2.4.1 Key molecules and signaling pathways regulating angiogenesis

Angiogenesis is considered a critical factor in restoring cardiac function during the repair process following MI (28, 40). Newly formed blood vessels not only supply the infarcted area with essential oxygen and nutrients but also support myocardial regeneration by increasing local oxygen availability, thereby improving cardiac function (52). The success of this process directly affects the recovery capacity of damaged myocardium.

Angiogenesis is tightly regulated by multiple molecular signaling pathways, among which vascular endothelial growth factor (VEGF) is one of the most critical regulators. VEGF binds to its primary receptor, VEGFR-2, and activates downstream signaling pathways such as the PI3K/Akt and MAPK pathways, promoting endothelial cell proliferation, migration, and survival (24). These endothelial cells subsequently form new vascular networks within the infarcted region to meet the blood supply demand of the injured myocardium.

#### 2.4.2 Dose-dependent effects of VEGF on angiogenesis and its therapeutic potential

VEGF is pivotal for post-myocardial infarction (MI) cardiac repair. Though angiogenic across concentrations, its efficacy is enhanced at ≥500 ng/mL (53); lower levels promote endothelial proliferation/migration but not the robust angiogenesis needed for repair (54). Optimizing VEGF concentration is thus critical to maximize its value in angiogenesis and cardiac function improvement.

Key studies confirm this: Shi et al. (54) used CBD-modified VEGF in rat MI, showing >30% infarct reduction, higher LVEF, less fibrosis, and mature vessels. Oduk et al. (55) delivered VEGF via nanoparticles, achieving higher local myocardial VEGF, ∼2-fold microvascular density, and faster contractile recovery. Zhou et al. (56) noted VEGF activates VEGFR2 to drive angiogenesis, improving LVEF by ∼20%.

These data highlight VEGF and its derivatives as promising for myocardial repair, with VEGF combined with nanoparticle delivery or co-therapeutics showing potential to boost cardiac function recovery.

#### 2.4.3 Mechanisms of other angiogenic factors

In addition to VEGF, fibroblast growth factor and platelet-derived growth factor (PDGF) are key contributors to angiogenesis. These factors increase endothelial and stromal cell (e.g., fibroblasts, smooth muscle cells, and pericytes) proliferation and migration through activation of the RAS/MAPK signaling pathway, thereby promoting the rapid expansion of the vascular endothelium and supporting stromal cells, which in turn accelerates angiogenesis and vessel maturation (57). In particular, PDGF stabilizes newly formed blood vessels by promoting smooth muscle cell and fibroblast proliferation, preventing the regression and rupture of nascent vessels.

#### 2.4.4 Role of the Notch signaling pathway in vessel branching

The Notch signaling pathway plays a critical role in vessel branching. The Notch1 receptor, which regulates endothelial cell differentiation and migration during angiogenesis, determines the branching pattern of blood vessels (24). When Notch1 signaling is activated, endothelial cells form more complex vascular branch structures, thereby increasing vascular coverage in the infarcted area. This process is essential for maintaining the stability and functionality of the vasculature.

#### 2.4.5 Innovative therapeutic strategies in preclinical studies

To further validate the roles of various molecules in MI repair, researchers have employed innovative therapeutic strategies in a range of preclinical animal models. For example, Mohindra et al. (58) utilized hyaluronic acid microrods to deliver the antifibrotic molecule decorin in a rat model of ischemia‒reperfusion MI. Compared with the control group, the decorin-treated group presented a significantly improved LVEF, with an increase of approximately 5.21% ± 4.29%, whereas decorin alone resulted in only a minor improvement of approximately −3.42% ± 1.86%. Additionally, this therapeutic strategy effectively reduces myocardial fibrosis and significantly decreases cardiomyocyte hypertrophy. These findings suggest that combining decorin with microrod carriers enables more stable local release of the drug into the heart, leading to enhanced therapeutic outcomes.

To further optimize treatment efficacy, some studies have combined VEGF with matrix-modifying factors to promote angiogenesis. Zhu et al. (59) used a novel biodegradable Dex-PCL-HEMA/PNIPAAm hydrogel combined with VEGF165 for injection in a rat model. They reported a significant increase in the VEGF165 concentration and upregulation of the VEGF receptors flk-1 and flt-1, which facilitated angiogenesis and reduced infarct size. Compared with the use of VEGF or hydrogel alone, the combination therapy significantly accelerated cardiac function recovery, indicating that the strategy of sustained release of VEGF165 effectively prolonged its therapeutic effect and enhanced cardiac repair efficiency.

These preclinical studies demonstrate that the combined use of bioactive factors, such as VEGF and decorin, with novel delivery systems, such as hydrogels or microrods, can not only effectively inhibit fibrosis but also promote angiogenesis, thereby creating a more stable microenvironment to support recovery of cardiac function. These innovative strategies provide important experimental evidence for personalized treatment of post-MI cardiac repair in the future. Research will continue to explore the synergistic effects of different growth factors, antifibrotic factors, and their signaling pathways in combination therapies, laying the foundation for the development of safer and more effective cardiac repair strategies (59).

## 3 Role of the Notch signaling pathway in post-MI repair

The Notch signaling pathway, an evolutionarily conserved cell-cell communication mechanism involved in cell fate determination, development, and tissue homeostasis, plays a crucial role in myocardial repair following (27). It exerts regulatory effects on the biological functions of multiple cell types (cardiomyocytes, vascular endothelial cells, fibroblasts) and contributes to core repair processes—including cardiac regeneration, angiogenesis, fibrosis modulation, and post-MI inflammatory responses—by influencing cell proliferation, differentiation, migration, and apoptosis (3). This involvement in inflammation is particularly critical, as it acts as a key link in coordinating the transition from tissue injury to repair, thereby comprehensively regulating cardiac remodeling progression (27). Moreover, the Notch pathway closely interacts with other key signaling pathways, such as the PI3K/Akt, TGF-β/Smad, Wnt/β-catenin, Hippo/YAP, VEGF, and Sonic Hedgehog pathways, to orchestrate orderly tissue repair and regeneration post-MI (15, 60–62).

### 3.1 Composition and activation mechanism of the Notch signaling pathway

**Figure 2:**
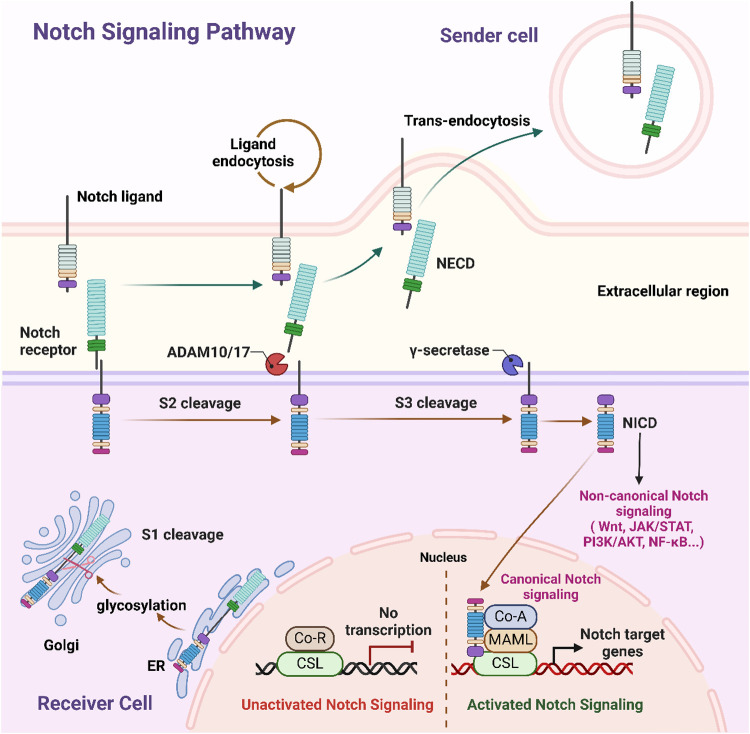
Schematic representation of canonical and non-canonical notch signaling pathways. This schematic representation depicts the communication of Notch signaling between the sending cell (presenting the Notch ligand) and the receiving cell (expressing the Notch receptor). In the sender cell, Notch ligand undergoes endocytosis, while on the receiver cell, ligand binding triggers sequential proteolytic cleavages of the Notch receptor: S2 cleavage by ADAM10/17, followed by S3 cleavage by γ-secretase, which releases NICD. NICD then translocates to the nucleus, where it drives canonical Notch signaling by forming a complex with CSL and co-activators (e.g., MAML) to activate Notch target gene transcription; it also mediates non-canonical Notch signaling via crosstalk with pathways like Wnt, JAK/STAT, PI3K/AKT, and NF-κB. Additionally, during receptor maturation in the receiver cell's Golgi and ER, glycosylation modulates Notch activity, and in the unactivated state, CSL associates with co-repressors (Co-R) to inhibit transcription. NECD, Notch extracellular domain; NICD, Notch intracellular domain; CSL, CBF1/suppressor of hairless/Lag1; ADAM, a disintegrin and metalloprotease; ER endoplasmic reticulum; Co-R, corepressor; Co-A, coactivator; MAML, mastermind-like. Figure created in BioRender.com.

The Notch signaling pathway is a highly conserved cell-to-cell communication pathway composed of core components, such as Notch receptors (e.g., Notch1 and Notch2), ligands (e.g., Jagged1, Delta-like), the γ-secretase complex, and the RBP-Jκ transcription factor (63). Upon binding of a Notch receptor to its ligand, the γ-secretase complex mediates cleavage and release of the Notch intracellular domain (NICD). The NICD subsequently translocates to the nucleus, where it activates the expression of target genes through its interaction with RBP-Jκ (Figure 2) (64, 65). Studies have shown that within a few hours post-MI, Notch1 signaling is activated in cardiomyocytes, endothelial cells, and fibroblasts, modulating oxidative stress, apoptosis, and inflammation to protect cardiac tissue (66).

Notch1 signaling enhances endothelial cell survival and angiogenesis through positive regulation of the PI3K/Akt pathway. Concurrently, the synergistic interaction between the Notch and TGF-β/Smad signaling pathways regulates fibroblast activation, thereby limiting excessive fibrosis (67). Consequently, crosstalk between the Notch, PI3K/Akt, and Hippo/YAP pathways is crucial for cardiomyocyte survival and proliferation (67, 68).

### 3.2 Role of Notch signaling in post-MI inflammation regulation

Notch signaling modulates post-MI inflammatory responses through multi-layered mechanisms, with effects exhibiting cell-type specificity and spatiotemporal duality—key to balancing injury clearance and tissue protection (27).

#### 3.2.1 Regulation of inflammatory cell phenotypes

Notch signaling directly influences the differentiation and functional polarization of core inflammatory cells. For macrophages (3), a key innate immune cell type in post-MI repair, Notch signaling shows distinct temporal effects: in the early post-MI phase, it is upregulated and promotes macrophage polarization toward the pro-inflammatory M1 phenotype via transcription factors like RBP-Jκ, a process that facilitates the phagocytic clearance of necrotic cardiomyocytes and tissue debris; accordingly, inhibition of Notch signaling (e.g., via gamma-secretase inhibitors) can attenuate such pro-inflammatory responses and reduce leukocyte infiltration. As the repair process transitions to the resolution phase, sustained Notch activation drives macrophages to switch toward the pro-reparative M2 phenotype, which in turn supports inflammation resolution and tissue repair.

#### 3.2.2 Modulation of cytokine and chemokine production

Activation of the Notch pathway fine-tunes the production of inflammatory cytokines and chemokines. This regulation ensures sufficient inflammatory responses for debris clearance while preventing excessive cytokine release that would exacerbate myocardial tissue damage (3).

#### 3.2.3 Promotion of inflammatory resolution

Timely resolution of inflammation is essential for transitioning from the injury phase to the proliferative phase of cardiac repair. Notch signaling contributes to this transition by suppressing persistent pro-inflammatory cascades and initiating reparative signaling, thereby creating a favorable microenvironment for subsequent tissue regeneration (62).

#### 3.2.4 Cell-type-specific effects beyond immune cells

Notch signaling also links inflammation to other post-MI repair processes through its regulatory effects on non-immune cells. In cardiac fibroblasts, Notch activation promotes cell proliferation and differentiation into myofibroblasts, establishing a direct connection between inflammatory signaling and fibrotic repair—an important link for balancing tissue repair and remodeling (3). In vascular endothelial cells, Notch modulates the expression of adhesion molecules such as VCAM-1 and ICAM-1; these molecules are critical for regulating leukocyte recruitment and trans-endothelial migration, which are key steps in the initiation and progression of post-MI inflammatory responses (62).

#### 3.2.5 Crosstalk with inflammation-related signaling pathways

The Notch pathway engages in extensive crosstalk with central inflammation- and remodeling-related pathways to coordinate repair. Notch can enhance NF-κB signaling to amplify the expression of pro-inflammatory cytokines (e.g., TNF-α, IL-6) during the early injury phase. Conversely, TGF-β reciprocally regulates the expression of Notch receptors, forming an integrated network that coordinates inflammatory responses and fibrotic remodeling (3, 62).

#### 3.2.6 Implications for therapeutic targeting

Due to its spatiotemporal duality and cell-type specificity, Notch represents a promising yet challenging therapeutic target for post-MI repair. Ideal strategies would aim to spatiotemporally modulate Notch activity—e.g., suppressing its early pro-inflammatory effects in monocytes while enhancing its later reparative functions in fibroblasts and macrophages (62, 69).

### 3.3 Role of Notch signaling in cardiomyocyte regeneration

While cardiomyocyte regeneration remains an ideal therapeutic goal for restoring cardiac function after myocardial infarction (MI), adult cardiomyocytes are mostly in a terminally differentiated state with significantly limited regenerative capacity—their proliferative activity is extremely low, making effective self-repair through regeneration difficult (70). This biological characteristic has gradually shifted the focus of current research toward “improving the cardiac microenvironment post-MI”: compared with solely pursuing cardiomyocyte regeneration, promoting effective repair and remodeling of myocardial tissue is the core direction for restoring cardiac function.

The core of modern therapeutic strategies revolves around “constructing a supportive cardiac microenvironment,” specifically through multi-target interventions (including anti-fibrotic, pro-angiogenic, and immunomodulatory approaches) combined with advanced technologies such as cell therapy, gene therapy, and biomaterials. These strategies improve cardiac structure and function from multiple dimensions, creating favorable conditions for myocardial repair (62).

Despite the limited regenerative capacity of adult cardiomyocytes, the Notch signaling pathway still plays a crucial role in myocardial repair: it not only regulates the “limited regenerative responses that occur” but also indirectly supports cardiac function recovery by optimizing the microenvironment.

Studies have shown that Notch1 signaling can promote cardiomyocyte proliferation by regulating the expression of cell cycle proteins (71). A typical case comes from zebrafish models: zebrafish cardiomyocytes possess strong regenerative capacity, and the Notch signaling pathway plays a decisive role in their regenerative potential—inhibiting Notch signaling significantly reduces the proliferative activity of cardiomyocytes and hinders myocardial regeneration, while activating Notch signaling markedly enhances cardiomyocyte proliferation and accelerates myocardial repair post-MI (15). This mechanism provides an important basis for understanding the “regulatory pathways of limited regeneration in adult cardiomyocytes.”

The Notch signaling pathway can also further amplify myocardial regeneration effects through interactions with other pathways, such as the Hippo/YAP and Wnt/β-catenin pathways (12, 72, 73). One study revealed that coactivation of the Notch and Wnt pathways significantly improves cardiomyocyte proliferation efficiency, thereby enhancing myocardial regenerative capacity (58, 64). Furthermore, the interaction with the Hippo/YAP pathway can inhibit the phosphorylation of YAP, promoting its nuclear translocation to regulate the expression of downstream regeneration-related genes, indirectly supporting myocardial repair (12).

Excessive fibrosis (caused by TGF-β-mediated fibroblast activation and collagen deposition) tends to occur in myocardial tissue post-MI. The scar tissue formed by fibrosis hinders myocardial regeneration and further impairs cardiac function (74). The Notch signaling pathway can reduce scar formation in myocardial tissue by downregulating TGF-β-mediated fibrotic responses—its mechanisms include inhibiting the differentiation of fibroblasts into myofibroblasts and reducing the deposition of collagen I/III, thereby creating a more suitable microenvironment for cardiomyocyte regeneration (even limited regeneration) (75, 76).

### 3.4 Regulation of angiogenesis by Notch signaling

Angiogenesis is a crucial process in cardiac repair following MI, and the Notch signaling pathway plays a key regulatory role in this process (61). In the early stages of MI, Notch1 signaling is activated in endothelial cells, promoting their proliferation and migration, which accelerates vascular regeneration in the infarcted area (77). Notch1 modulates vessel branching and endothelial cell lumen formation through its interaction with VEGF signaling, thereby increasing the density and stability of the vascular network (78, 79). Moreover, the synergistic effects of Notch and other signaling pathways, such as the PI3K/Akt and RAS/MAPK pathways, significantly contribute to angiogenesis. For example, the PI3K/Akt pathway enhances the effects of Notch signaling on angiogenesis by promoting endothelial cell survival and migration (52, 61). Additionally, the Notch and Hippo signaling pathways coordinate the spatial and temporal regulation of vascular formation, differentiation, and homeostasis, playing pivotal roles in maintaining the stability of newly formed vessels (56, 80–82).

Studies have shown that the inhibition of Notch signaling impairs angiogenesis, whereas its activation promotes repair in the infarcted area by increasing endothelial cell proliferation and differentiation (67). Through these mechanisms, Notch signaling contributes to effective vascular regeneration, ultimately improving the blood supply to the damaged myocardium and supporting cardiac function recovery.

### 3.5 Regulation of cardiac fibrosis and scar formation by Notch signaling

Fibrosis and scar formation are inevitable processes in cardiac repair after MI. However, excessive fibrosis can severely impair cardiac function (83). The Notch signaling pathway plays a dual regulatory role in fibrosis by modulating fibroblast proliferation, differentiation, and migration (84). According to Gude and Sussman (15), Notch signaling plays a critical role in the repair of adult cardiac injuries by regulating the proliferation and differentiation of specific cardiomyocytes, thereby contributing to tissue reconstruction and repair. MacGrogan et al. (85) reviewed the complex interactions between Notch and other signaling pathways, highlighting its multifaceted role in both cardiac fibrosis and regeneration, including its ability to regulate the differentiation and proliferation of various cardiac cell types. Xiao et al. (86) reported that Notch signaling can inhibit excessive proliferation of cardiac fibroblasts by modulating the TGF-β1/α-SMA/collagen pathway, thereby exerting an inhibitory effect on fibrosis. These studies suggest that Notch signaling can either promote or inhibit fibrosis, depending on specific activation patterns and the pathological environment of the heart.

Inhibition of Notch signaling not only exacerbates fibrosis but also disrupts scar formation and remodeling (83). For example, one study revealed that inhibition of Notch1 signaling leads to increased cardiac fibrosis, thinning of the ventricular wall, and excessive scar tissue formation (76). Conversely, moderate activation of Notch signaling can promote the controlled migration and proliferation of fibroblasts and myofibroblasts, supporting balanced scar formation that stabilizes cardiac structure and function.

Interactions between Notch signaling and pathways such as the Hippo/YAP and TGF-β pathways are particularly critical in the regulation of fibrosis (87–90). These pathways coordinate during myocardial fibrosis to regulate fibroblast activation and scar tissue formation, ultimately reducing fibrosis progression and promoting recovery of cardiac function recovery (91–93).

## 4 Therapeutic strategies targeting the Notch signaling pathway

The Notch signaling pathway plays a crucial role in the regulation of key physiological processes, such as cardiomyocyte proliferation, differentiation, apoptosis, and fibrosis. Thus, it has great potential for cardiac repair following MI. Modulating this pathway can provide effective therapeutic strategies to promote cardiac regeneration post-MI (94). Increasing evidence suggests that the Notch pathway has significant regulatory effects on multiple cell types, including cardiomyocytes, fibroblasts, and vascular endothelial cells (95, 96).

### 4.1 Potential drug development targeting Notch

With a deeper understanding of the pivotal role of the Notch signaling pathway in cardiac repair, the development of drugs targeting this pathway has become a research focus. Activation of Notch1 signaling has been shown to effectively reduce cardiac fibrosis, enhance cardiomyocyte survival, and improve overall cardiac function. For example, Kratsios et al. demonstrated that Notch1 activation significantly reduced fibrotic responses post-MI and enhanced cardiac regeneration through antiapoptotic mechanisms. Further research revealed that overexpression of Jagged1 not only promoted the proliferation of cardiac progenitor cells but also played a vital role in alleviating cardiac hypertrophy and fibrosis (83).

In the field of drug development, γ-secretase inhibitors have been extensively studied as key inhibitors that target the Notch signaling pathway. By inhibiting the activity of this enzyme, the excessive progression of myocardial fibrosis can be effectively reduced. Studies have shown that γ-secretase inhibitors (e.g., DAPT) can significantly decrease the extent of fibrosis after MI while improving cardiac function (18).

### 4.2 Inhibitors and activators of the Notch signaling pathway

The Notch signaling pathway has different regulatory functions under various pathological conditions. During cardiac repair, the activation and inhibition of the Notch pathway play distinct physiological roles. Activation of the Notch1 pathway promotes cardiomyocyte survival and angiogenesis. Research has demonstrated that Notch1 agonists not only protect cardiomyocytes through antiapoptotic mechanisms but also significantly increase endothelial cell migration and capillary formation, thereby further promoting the restoration of blood flow in ischemic regions (96).

Conversely, inhibition of the Notch signaling pathway has significant clinical value. Studies have shown that γ-secretase inhibitors can effectively suppress myocardial fibrosis and reduce fibroblast activity (95). Additionally, different Notch ligands, such as Jagged1 and Dll4, play different roles in cardiac repair. Jagged1 mainly improves regenerative capacity by promoting cardiomyocyte proliferation, whereas Dll4 regulates angiogenesis. Excessive activation of Dll4 may inhibit capillary formation, indicating its role in negatively regulating certain aspects of blood vessel development (97).

### 4.3 Gene therapy and RNA interference techniques

Gene therapy and RNA interference (RNAi) technologies provide powerful tools for targeting the Notch signaling pathway (98). Using gene transfer techniques, specific Notch signaling molecules, such as the Notch1 receptor or its downstream effectors Hes1 and Hey2, can be activated or inhibited following myocardial injury, thereby promoting cardiac regeneration (99). Studies have demonstrated that delivering the active form of Notch1 (NICD1, the Notch1 intracellular domain) into cardiomyocytes via adeno-associated viral vectors can effectively reduce fibrosis and enhance cardiac function (99). Moreover, the upregulation of effectors, such as Hes1, can significantly inhibit cardiomyocyte apoptosis and fibrosis, further promoting cardiac repair and functional recovery.

RNA interference techniques, such as small interfering RNAs (siRNAs) and microRNAs, have also demonstrated precise regulatory effects on the Notch signaling pathway. For example, miR-199b can increase VEGF secretion and promote angiogenesis by inhibiting Jagged1 expression (100). Additionally, research has shown that targeting Notch1 with siRNA can effectively reduce myocardial fibrosis and inflammatory responses, thereby protecting cardiomyocytes (95).

### 4.4 Stem cell therapy and Notch regulation

Stem cell therapy shows great promise in the field of myocardial regeneration, and the Notch signaling pathway plays a critical role in regulating the differentiation and function of stem cells. A study by Zheng et al. (60). demonstrated that activation of the Notch1 signaling pathway enhances the angiogenic capacity of stem cells. The mechanism underlying this effect involves the upregulation of VEGF and downstream Notch effectors such as Hes1, thereby promoting tissue repair after MI. Additionally, the Notch pathway regulates the proliferation and differentiation of bone marrow-derived endothelial progenitor cells, particularly through interactions between Notch1 and VEGFR2, which in turn facilitates vascular regeneration in ischemic regions (46).

Regulation of the Notch pathway is particularly important during the formation of new blood vessels. By enhancing the proliferation of endothelial progenitor cells and modulating key molecules, such as the DLL4-Notch ligand and its crosstalk with the VEGF signaling pathway, Notch signaling improves the blood supply to ischemic areas, effectively repairing myocardial tissue (101). The interactions between these signaling molecules during angiogenesis further illustrate that Notch regulation is crucial for microvascular stability and improved blood supply following MI.

Therefore, combining Notch signaling modulation with stem cell therapy holds significant potential for providing more precise and efficient therapeutic strategies for patients with MI. This combination approach could pave the way for innovative treatment options that optimize myocardial repair and functional recovery.

## 5 Problems that need to be addressed

### 5.1 Mechanistic understanding

Regarding the mechanistic understanding of Notch signaling in cardiac repair, two critical aspects require further in-depth investigation. First, the precise interactions between Notch signaling and other pivotal cardiac repair pathways—specifically PI3K/Akt, Wnt/β-catenin, and TGF-β—need better characterization (62), as clarifying these pathway crosstalks is essential for developing effective combination therapies that target cardiac repair mechanisms. Additionally, the differential roles of Notch signaling in various cardiac cell types, including cardiomyocytes, endothelial cells, fibroblasts, and immune cells, demand comprehensive elucidation (102); understanding such cell-type-specific mechanistic roles is fundamental to deciphering how Notch signaling orchestrates overall cardiac repair processes at the cellular and molecular levels.

### 5.2 Therapeutic development

In the context of therapeutic development for Notch signaling-based cardiac interventions, two key unmet needs and challenges stand out prominently, as they directly hinder the translation of preclinical insights into safe and effective clinical treatments. First, the development of cardiac-specific Notch modulators capable of circumventing systemic toxicity represents a major unmet requirement (15, 103): current Notch-modulating approaches lack the necessary tissue specificity to avoid off-target effects in non-cardiac organs, which compromises their safety for clinical application and limits their therapeutic potential. Second, there is an urgent need for advanced delivery systems that can achieve cardiac-specific targeting while preserving therapeutic efficacy (104) of Notch agonists or inhibitors. Although nanoparticle-based delivery platforms have shown promising potential in this regard—demonstrating initial capacity to concentrate agents in cardiac tissue—they still require further optimization to overcome technical barriers (e.g., low bioavailability, inconsistent targeting efficiency) and meet rigorous clinical standards for reproducibility and safety.

Against this backdrop of unmet needs, nanotechnology has emerged as a powerful tool to enhance therapeutic targeting in Notch-based cardiac interventions, directly addressing the delivery and specificity challenges outlined above. By enabling the precise delivery of Notch agonists or inhibitors to damaged myocardial regions, nanotechnological platforms minimize systemic exposure and associated side effects. For instance, the delivery of Notch1 agonists via nanoparticles significantly enhances myocardial regeneration, reduces fibrosis by more than 50%, and notably increases angiogenesis (105). Additionally, combination therapy strategies are being advanced to amplify therapeutic efficacy: coadministration of a Notch1 agonist with a PI3K/Akt pathway activator has been shown to synergistically promote myocardial cell survival and regeneration, resulting in significant improvements in cardiac function recovery (106). Together, these innovations directly tackle the core unmet needs in therapeutic development, bridging gaps between preclinical potential and clinical applicability.

Beyond these nanotechnological and combination therapy advances, a promising frontier in therapeutic development lies in integrating the multitarget mechanisms of traditional Chinese medicines (TCMs) with cutting-edge biotechnologies such as gene editing and nanotechnology, to refine Notch signaling regulation, particularly in antiangiogenic research. TCMs, with their inherent capacity to modulate multiple biological pathways simultaneously, offer a unique advantage for addressing the complexity of Notch signaling networks, which often interact with other pathways in cardiac disease. Equally critical is the interplay between the Notch and VEGF pathways: in both cancer therapy and cardiovascular disease treatment, this pathway crosstalk governs key processes like angiogenesis and vascular remodeling, making it a critical focus for future targeted therapies. By deepening the exploration of TCM compounds (to leverage their multitarget properties) and applying modern biotechnologies (to enhance precision), there is considerable potential to develop more precise, effective, and well-tolerated treatment strategies—ultimately offering new solutions for addressing complex diseases in clinical practice and advancing the therapeutic development of Notch-targeted interventions.

### 5.3 Clinical translation

In the context of clinical translation for Notch-targeted cardiac therapies, multiple critical gaps and challenges demand urgent attention to bridge the divide between preclinical research and real-world clinical application. First, the identification and validation of reliable biomarkers to predict patient response to these Notch-targeted interventions are essential for achieving meaningful clinical success (70); current approaches, however, lack effective patient stratification strategies—a limitation that not only hinders the ability to tailor treatments to individuals most likely to benefit but also increases the risk of suboptimal outcomes and wasted clinical resources. Additionally, the establishment of standardized regulatory pathways and frameworks specifically designed for evaluating Notch-targeted cardiac therapies is critically needed (107); clear, consistent regulatory guidelines are foundational to streamlining the clinical development process, ensuring rigorous assessment of safety and efficacy, and ultimately enabling the smooth transition of promising Notch-based interventions from laboratory research to routine clinical practice.

Beyond these translational gaps, the multifaceted roles of the Notch signaling pathway—coupled with its context-dependent complexities across different tissues and cell types (108)—pose another major hurdle: while Notch1 has been shown to promote myocardial regeneration and reduce fibrosis, excessive activation of Notch3 in vascular smooth muscle cells is closely associated with the progression of atherosclerosis (109); moreover, different Notch ligands play distinct roles in cardiac repair, such as Jagged1 [which binds to Notch1 to promote myocardial cell regeneration, activate endothelial cell proliferation, and enhances angiogenesis (109)] vs. overactivated Dll4 (which can inhibit the VEGF signaling pathway, leading to abnormal vascular formation and impaired myocardial repair). Consequently, the precise modulation of the Notch pathway under varying pathological conditions remains a significant challenge for clinical translation, and despite extensive preclinical research, clinical evidence for Notch-targeted cardiac therapies remains extremely limited—with no Notch-specific therapies having received regulatory approval for MI treatment to date (103).

## 6 Future perspectives and emerging solutions

### 6.1 Advanced therapeutic strategies

In terms of advanced therapeutic strategies for Notch-related cardiac interventions, two promising directions have emerged to overcome existing limitations and enhance treatment efficacy. First, precision medicine approaches emphasize the development of patient-specific Notch modulation strategies, which are tailored based on individuals' unique genetic and epigenetic profiles (70); this personalized approach holds significant potential to address the context-dependency challenges that currently restrict the therapeutic effectiveness of Notch-targeted interventions, ensuring treatments align with the distinct biological characteristics of each patient. Second, combination therapies focus on the integration of Notch modulation with other cutting-edge regenerative approaches, encompassing growth factor delivery, tissue engineering, and immunomodulation (62); by leveraging the synergies between Notch signaling and these complementary strategies, such combinations aim to create more comprehensive and potent therapeutic regimens that go beyond the capabilities of single-target interventions.

### 6.2 Technological innovations

In the realm of technological innovations for Notch-associated cardiac therapies, both current impactful advancements and future research directions are driving progress in targeted, regenerative, and precise treatment approaches for cardiac conditions. One key current innovation centers on the development of advanced nanoparticle systems endowed with cardiac-specific targeting capabilities (104); these engineered delivery platforms are designed to minimize systemic exposure of therapeutic agents—thus reducing off-target effects—while simultaneously maximizing their concentration and therapeutic efficacy within the heart, addressing a longstanding technical challenge in precise drug delivery for cardiac interventions. Another notable technological breakthrough stems from the integration of Notch pathway modulation with cardiac tissue engineering approaches (103); this synergistic combination unlocks novel possibilities for cell replacement therapy, as it merges the regulatory role of Notch signaling in guiding cell fate (e.g., proliferation, differentiation of cardiac progenitor cells) with the structural support and functional restoration potential of tissue engineering platforms, thereby expanding the scope and effectiveness of regenerative strategies for treating cardiac damage. Beyond these existing advancements, future research is poised to further optimize Notch signaling regulation through cutting-edge technologies. CRISPR/Cas9 gene editing technology, for example, has already been utilized to precisely modulate the Notch pathway, with studies demonstrating that knocking out Notch3 can effectively slow the progression of atherosclerosis and reduce myocardial fibrosis (75). Additionally, RNAi has shown promising potential for targeted regulation, as it can silence Notch3 gene expression via siRNA, thereby reducing vascular inflammation and improving myocardial repair outcomes. Together, these current and emerging technological innovations collectively advance the development of more effective, specific, and regenerative Notch-associated cardiac therapies.

### 6.3 Regulatory and clinical development

Two critical strategies are being advanced to accelerate their safe and effective translation into clinical practice, while significant safety challenges—rooted in the pathway's broad physiological roles—must also be addressed. First, improved trial designs—characterized by enhanced frameworks that incorporate biomarker-driven patient stratification and optimized timing protocols (70)—represent a key advancement; by ensuring that trials enroll patients most likely to respond to treatment and administer therapies at biologically optimal timepoints, these designs not only align with regulatory expectations for rigorous evidence generation but also have the potential to significantly improve the success rate of clinical translation, reducing the risk of trial failures due to poor patient selection or suboptimal dosing timing. Complementing this, safety optimization efforts focus on developing intermittent dosing schedules and reversible Notch modulators (62); these innovations are tailored to minimize off-target toxicity and long-term adverse effects—critical considerations for regulatory approval—while preserving the therapeutic efficacy of Notch targeting, addressing a historical barrier that has hindered Notch-based cardiac therapies from advancing through the regulatory pipeline. However, a pivotal challenge in these safety and regulatory efforts lies in the Notch signaling pathway's critical roles across multiple physiological systems, as targeting it may induce widespread side effects. For instance, γ-secretase is a key enzyme in Notch activation: while γ-secretase inhibitors (e.g., DAPT) effectively inhibit the Notch pathway, long-term inhibition can cause adverse effects, including interfering with the cleavage of amyloid precursor protein, which may contribute to Alzheimer's disease-related pathology (109), and disrupting the regulation of systemic stem cells, leading to multi-organ side effects such as skin dryness and hyperkeratosis, as well as negative impacts on the liver, kidneys, and immune system (110). These safety concerns thus remain a major bottleneck in the regulatory and clinical development of Notch-targeted cardiac therapies, underscoring the necessity of the aforementioned trial design and safety optimization strategies to balance efficacy with regulatory compliance and patient safety.

## 7 Conclusion and future perspectives

Targeted regulation of the Notch signaling pathway offers revolutionary potential for cardiovascular disease treatment, particularly in promoting tissue repair following MI. Despite the significant therapeutic effects observed, the multifunctionality of the Notch pathway across various cell types presents challenges for its precise clinical translation. Moreover, therapies targeting Notch signaling must address issues related to side effects and long-term safety.

Future research will focus on personalized treatment and target optimization. Technologies such as gene editing, RNA interference, and nanoparticle drug delivery have provided robust support for the advancement of notch-targeted therapies. As these innovative technologies continue to evolve, the therapeutic application of the Notch signaling pathway holds promising prospects and is expected to provide more precise and effective solutions for cardiovascular disease treatment in the future.
